# Abnormal Leg Muscle Latencies and Relationship to Dyscoordination and Walking Disability after Stroke

**DOI:** 10.1155/2011/313980

**Published:** 2010-12-29

**Authors:** Janis J. Daly, Kristen Roenigk, Roger Cheng, Robert L. Ruff

**Affiliations:** ^1^Research Service 151-W, Louis Stokes Cleveland VA Medical Center, 10701 East Boulevard, Cleveland, OH 44106, USA; ^2^Department of Neurology, School of Medicine, Case Western Reserve University, Cleveland, OH 44106, USA

## Abstract

The purpose was to determine timing characteristics of leg muscle latencies for patients following stroke (>12 months) who had persistent coordination and gait deficits, and to determine the relationships among abnormal latencies, dyscoordination, and gait deficits. We compared nine healthy controls and 27 stroke survivors. Surface electromyography measured activation and deactivation latencies of knee flexor and extensor muscles during a ballistic knee flexion task, consistency of latencies across repetitions, and close coupling between agonist and antagonist muscle latencies. We measured Fugl-Meyer (FM) coordination and the functional gait measure, six minute walk test (6MWT). For stroke subjects, there were significant delays of muscle activation and deactivation, abnormal inconsistency, and abnormal decoupled agonist and antagonist activations. There was good correlation between activation latencies and FM and 6MWT. Results suggest abnormal timing characteristics underlie coordination impairment and dysfunctional gait. These abnormal muscle activation and deactivation timing characteristics are important targets for rehabilitation.

## 1. Introduction

Lower limb coordination deficits persist after stroke rehabilitation. Timing characteristics of muscle activation are integral to coordinated movement and normal gait. Though abnormal timing characteristics for upper limb dyscoordination have been reported, there is little information regarding the nature of lower limb muscle activation abnormalities and their relationship to coordinated movement and functional gait. Because the mechanisms underlying coordination impairments and gait deficits are poorly understood, it is difficult to accurately target rehabilitation interventions. The purpose of this study was twofold. First, we identified the abnormal timing characteristics of muscle activation and deactivation for knee flexor and extensor muscles for patients following stroke who had persistent coordination and gait deficits in the swing phase of gait. Second, we determined the association between muscle activation latency and coordination impairment and dysfunctional gait. 

In the swing phase of gait, complex and precisely coordinated muscle activation and deactivation timings are critical for safe, coordinated walking. There are relatively few studies that characterize in stroke survivors, the precision of muscle activation and deactivation latencies that are demanded during gait-swing phase. In an attempt to study flexion and extension limb movements, one study utilized a cycle pedaling task to study muscle activation relationships during mechanically coupled movements of right and left lower limbs. This study showed delayed activation of the paretic vastus medialis during cycling limb extension, and early activation and termination of hip and knee flexors [1]. There is controversy, however, with regard to whether or how significantly these observations are related to functional gait. That is, some have shown that functional gait gains occurred without any significant change in the abnormal electromyography (EMG) durations [2, 3], whereas another study reported that neurological impairment was not necessary in creating some of these abnormal EMG patterns [4]. 

An alternative way to study impairment of the coordinated components of gait-swing phase, would be to begin with the study of the coordination of the timing of a simple, single joint movement that is required in gait-swing phase. During swing phase, there is demand for coordinated muscle latencies for knee flexion in order for the limb to clear the floor normally in the sagittal plane. After stroke, a “stiff-legged” pattern is characteristic, for which knee flexion coordination is abnormal, and movement excursion is significantly less than normal throughout swing phase [5, 6]. Not only does the stiff-legged gait pattern exhibit absence of timely execution of swing phase limb flexion, but also, there is highly variable limb flexion excursion [7] and inability to cease the performance of the limb extension pattern even when hip, knee, and ankle flexor muscle strength is adequate [8].

In order to adequately treat swing phase gait deficits, it is important to understand the coordination deficits in knee flexion and extension movements. Coordination is based on the precise coordination of muscle activation and deactivation latencies at a given joint, for the contracting muscle (agonist), as well as the lengthening muscle (antagonist). This coordination appears impaired in stroke survivors for coordinated knee movements. Therefore, the first purpose of this study was to identify, for stroke survivors with persistent swing phase deficits, timing characteristics of knee flexor and extensor muscles during a single joint knee flexion task. The timing characteristics studied were as follows: knee flexor and extensor muscle activation and deactivation latencies, consistency of activation latencies during repeated knee flexion movements, and the relative timing of agonist/antagonist activation during a ballistic knee flexion task. The second purpose was to characterize the relationships among abnormal delay in muscle activation latency, joint movement coordination impairment, and dysfunctional gait.

## 2. Materials and Methods

### 2.1. Subjects

For group one, twenty-seven subjects were enrolled who were more than 12 months following stroke. They had persistent swing phase gait deficits, including absent or attenuated knee flexion during swing phase. For group two, nine healthy adult control subjects were enrolled who had no known neurological diagnoses and a normal gait-swing phase. The study was conducted according to the Declaration of Helsinki and oversight of the Human Subjects' Protection Board of the Medical Center. Subjects were informed of the study prior to participation and provided written informed consent.

### 2.2. Experimental Procedures and Measures

#### 2.2.1. EMG Data Acquisition and Analysis

**Figure 1:**
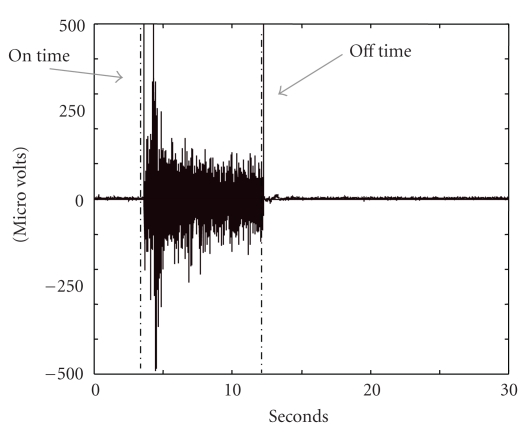
Raw EMG signal with calculated muscle on and off times superimposed.

Using an I410 data acquisition system by J & J Engineering, Inc. (Poulsbo, WA), electromyographical (EMG) activity of the long head of the biceps femoris (LHB) and the vastus medialis (VM) was recorded at 1100 Hz sampling frequency (band-pass filter = 40–360 Hz with a 1 pole Chebyshev filter) with active bipolar surface electrodes (silver/silver chloride strips 1 cm square and 1 cm apart, along the longitudinal axis of the muscle). Further, EMG signals were rectified and determination was made regarding the presence or absence of onset of muscle activation. Muscle activation “onset” was identified when, for a 100 ms period, the EMG signal surpassed and remained above 1 standard deviation of the baseline activity recorded at rest [9, 10]. We visually checked the calculated muscle activation onset time for each trial by superimposing the calculated activation onset onto the raw data. Visual analysis agreed with the software identification of onset time (example, Figure 1).

#### 2.2.2. Motor Task Performance

Muscle activation latencies were recorded for two knee flexion motor tasks: (1) volitional knee flexion while in the side-lying position, with the hip in neutral position (0° flexion), and (2) volitional knee flexion while in the standing position with all weight on the uninvolved leg, with the involved hip in neutral, the involved knee flexed so that the toe rested on the floor behind the subject, and the ankle in neutral position (0° plantarflexion or dorsiflexion). Motor task 1 was chosen as the most simple condition of knee flexion (gravity eliminated from the task). Motor task 2 was chosen because it represents a static test condition of knee flexion movement against gravity in the standing, upright position, with the limbs and torso in the same position as during the knee flexion movement that is performed in the dynamic functional task of walking. 

For the side-lying knee flexion task, the subject was stabilized in a side-lying position on a mat, lying on the uninvolved side, with the involved limb supported on a platform parallel to the mat surface. The thigh was stabilized in a position of 0° of hip flexion. The shank and foot were secured to a low-friction skate, so that knee flexion could be easily performed in a horizontal plane. The knee was positioned in full knee extension, prior to the test. For the standing test, the subject was stabilized by resting the uninvolved forearm on a secure surface while grasping an attached bar with the uninvolved hand.

The command to move was communicated to the subjects using a buzzer analog signal that was activated for 10 seconds and recorded simultaneously with the EMG signal. The subjects were instructed to flex the knee as quickly as possible when the analog command signal was activated, hold the maximum knee flexion position for 10 seconds until the buzzer turned off, and then immediately relax as completely as possible. Data was collected for ten seconds baseline prior to the command to move, and during the resting baseline time, the EMG signal was monitored to ensure that the subject's muscles were in the relaxed state. For each of the two motor tasks, we collected data during ten trials for each subject.

#### 2.2.3. Measures of Coordination and Functional Walking

Coordination of lower limb isolated joint movement control was assessed using the Fugl-Meyer coordination scale (FM) subtest for the lower extremity. The FM is a 34-point ordinal scale that assigns a score according to ability to volitionally move at one joint either simultaneously with or independent of total limb flexor or extensor synergistic patterns. The FM is a sensitive, reliable, and valid measure of the coordination of isolated joint movement coordination after stroke [11–14].

Functional walking was assessed using an index of walking speed, the six minute walk test (6MWT, distance walked per 6 minutes). For healthy adults, the 6MWT was significantly correlated with energy expenditure *r* = .63; *P* < .001; [15]), activities of daily living [16], and quality of life [17]. The 6MWT was reliable and valid for use in both healthy adults and stroke patients [18, 19].

### 2.3. Data Analysis

#### 2.3.1. Muscle Activation and Deactivation Latency Comparisons for Hemiparetic versus Control Subjects

Because of heterogeneous variances between the two groups, comparisons between hemiparetic and controls for activation and deactivation latencies were made using the nonparametric Kruskal-Wallis test and Bonferroni adjustments for multiple tests [20, 21].

#### 2.3.2. Consistency of Muscle Activation Latencies for a Repeated Task

Consistency was defined as the difference between the activation latency for a muscle for a single trial and the mean latency across trials for that muscle for a given task. Consistency was calculated for each subject, for each motor task according to the following formula:

(1) $$\text{Consistency}=\left|\left(\overset{®}{x}-x_{i}\right)\right|,$$

where $\overset{®}{x}$ is the mean of the onset latencies for one muscle, during a specific task, for one subject; *x*_*i*_ is the onset latency for the single trial, “*i*”, for that subject, during that task, for that muscle.

Comparisons between hemiparetic and control subjects were made as described in the section above.

**Close Coupling of Agonist and Antagonist Muscles**

Close coupling was defined as the absolute time difference between muscle activation onset latency for the agonist (the flexor muscle that produced the flexion movement) and the antagonist (the extensor muscle that can oppose the flexor movement). Close coupling was calculated for each trial during a particular motor task according to the following formula:

(2) $$\text{Close-coupling}=\left|x_{\text{antagonist},i}-x_{\text{agonist},i}\right|,$$

where *x*_antagonist,*i*_ is the muscle activation onset latency for the antagonist for a single trial, “*i*”, during a particular motor task for a particular subject. *x*_agonist,*i*_ is the muscle activation onset latency for the agonist for the same trial during the same task for the same subject. Comparisons between hemiparetic and control subjects were made as described above.

**Relationship between Activation Latency and Both Coordination Impairment and Functional Gait**

A Spearman correlation for nonparametric measures [22] was conducted to determine the correlation values between muscle activation latency and both the FM coordination scale and the 6MWT functional walking index.

## 3. Results

Subjects ranged in age from 40–77. The range of FM coordination scores was 11–28. The range of results for the 6 MWT was 14.6 m–323.1 m. Other subject characteristics are provided in Table 1. 

### 3.1. Activation Latencies 

#### 3.1.1. Task 1

**Figure 2:**
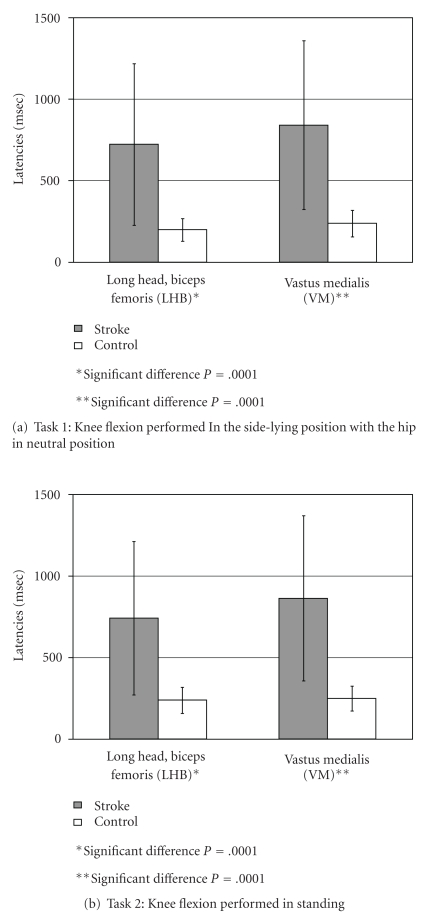
Comparison of onset latencies for control and stroke subjects.

For task 1, healthy controls showed short duration activation latencies for both LHB (200 ± 79 ms) and VM (239 ± 81 ms; Figure 2(a), white bars, resp.). In contrast, for each of the two knee flexion tasks, respectively, there was a significant activation delay for hemiparetic subjects (Figure 2, shaded bars) for the long head of the biceps femoris (LHB; 723 ± 496 ms) and the vastus medialis (VM; 841 ± 517 ms). For the task 1 comparison between stroke and control, there was a significant difference for stroke versus controls for mean activation latency as follows: (1) LHB muscle (*P* = .0001) and (2) VM muscle (*P* = .0001).

#### 3.1.2. Task 2

For task 2, healthy controls exhibited short-duration activation latencies for both LHB and VM muscles (Figure 2(b), white bars, resp.). For the task 2 comparison (Figure 2(b)) between stroke and control, there was a significant difference for stroke versus controls for mean activation latencies as follows: (1) LHB muscle: controls, 239 ± 81 ms versus hemiparetic, 743 ± 471 ms (*P* = .0001); (2) VM muscle: controls, 250 ± 77 ms versus hemiparetic, 864 ± 506 ms (*P* = .0001).

### 3.2. Muscle Deactivation Latencies

**Figure 3:**
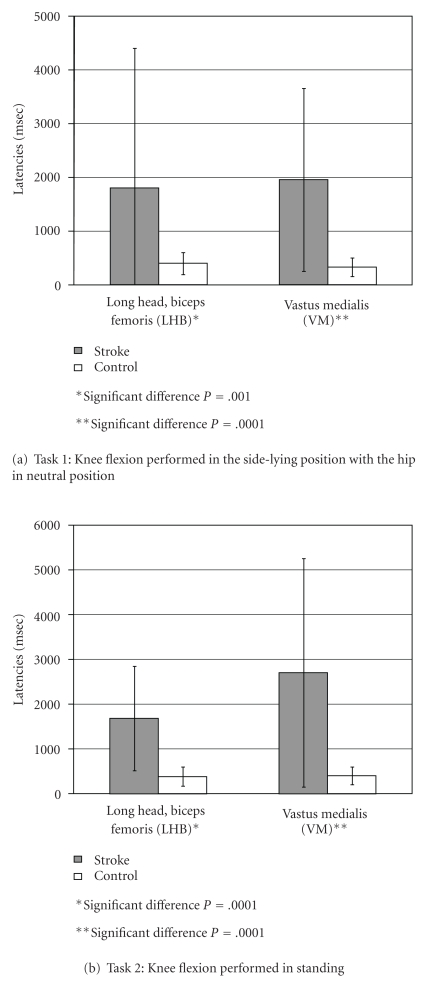
Comparison of deactivation latencies for control and stroke subjects.

For the two tasks shown in Figures 3(a) and 3(b), healthy controls showed relatively short deactivation latencies averaging less than 403 ms. Figures 3(a) and 3(b) illustrate for each task, respectively, that there was a significant deactivation delay for hemiparetic subjects (shaded bar) versus controls (white bars) for the LHB and the VM.

#### 3.2.1. Task 1

For the task 1 comparison between stroke and control, there was a significant difference for mean deactivation latency as follows: (1) LHB muscle: controls, 401 ± 204 ms versus hemiparetic, 1802 ± 2601 ms (*P* = .001); (2) VM muscle: controls, 331 ± 173 ms versus hemiparetic, 1957 ± 1701 ms (*P* = .0001).

#### 3.2.2. Task 2

For the task 2 comparison between stroke and control, there was a significant difference for stroke versus controls for mean deactivation latency as follows: (1) LHB muscle: controls, 386 ± 218 ms versus hemiparetic, 1681 ± 1166 ms (*P* = .0001); (2) VM muscle: controls, 403 ms ± 198 ms versus hemiparetic, 2704 ± 2555 ms (*P* = .0001).

### 3.3. Consistency during Repetitive Performance of a Simple Motor Task

**Figure 4:**
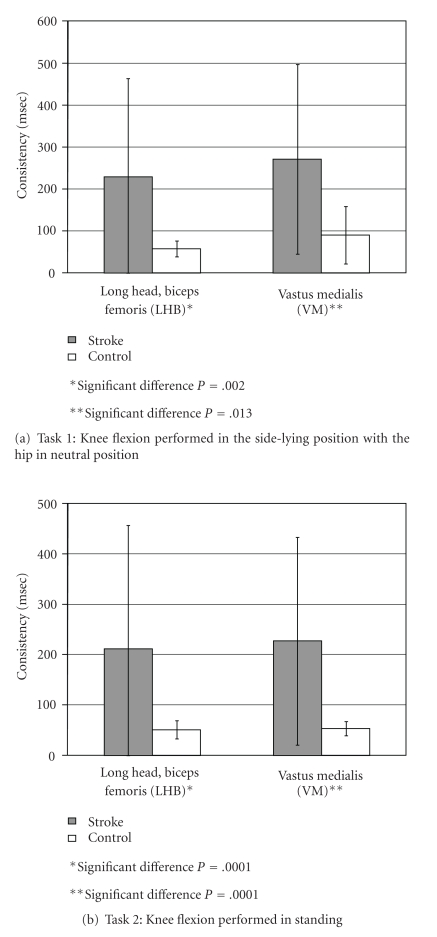
Comparison of consistency for control and stroke subjects.

For the two tasks shown in Figures 4(a) and 4(b), healthy controls showed high consistency of activation averaging a consistency within 90 ms (white bars, resp., Figure 4).

#### 3.3.1. Task 1

Figure 4(a) illustrates that for task 1, there was a significant difference between hemiparetic and control subjects regarding consistency of muscle activation for each muscle. Mean consistency and test results for the two muscles were as follows: (1) LHB muscle: controls, 57 ± 19 ms versus hemiparetic, 228 ± 233 ms (*P* = .002); (2) VM muscle: controls, 90 ± 68 ms versus hemiparetic, 271 ± 226 ms (*P* = .013).

#### 3.3.2. Task 2

Figure 4(b) shows that for task 2 there was a significant difference between hemiparetic and control subjects regarding consistency of muscle activation for each muscle. Mean consistency and test results were as follows: (1) LHB muscle: controls, 51 ± 18 versus hemiparetic, 211 ± 245 (*P* = .0001); (2) VM muscle: controls, 53 ± 14 ms versus hemiparetic, 227 ± 206 ms (*P* = .0001).

### 3.4. Close Coupling between Agonist and Antagonist Activation Onset

#### 3.4.1. Task 1

**Figure 5:**
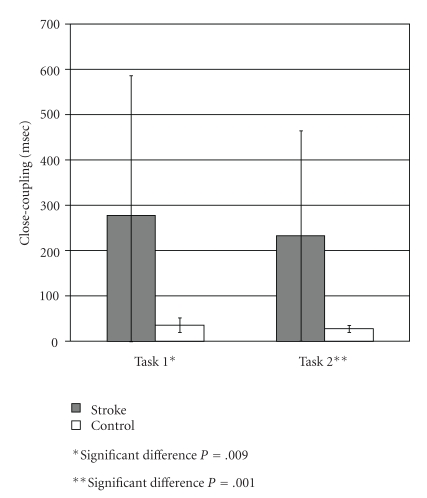
Comparison Of Close-Coupling For Stroke And Control Subjects.

For task 1 (Figure 5), controls had close-coupling values ranging 23–70 msec (mean = 35 ± 16 msec). Whereas, hemiparetic subjects had values ranging 20–1,530 msec (mean = 277 ± 308 msec). For task 1, there was a significantly longer interval between agonist and antagonist activation for stroke versus control subjects (Figure 5; *P* = .009). Eighty-five percent of stroke subjects (23/27) had close-coupling values exceeding the range of the control subjects (>70 msec).

#### 3.4.2. Task 2

For task 2, control subjects had close-coupling values of 20–40 msec (mean = 27 ± 8 msec). Whereas, hemiparetic subjects had values ranging from 20 to 1,076 msec (mean = 233 ± 232 msec). For task 2, there was also a significantly longer interval between agonist and antagonist activation for stroke versus control subjects (Figure 5; *P* = .001). Eighty-eight percent had close-coupling values beyond the range of the control subjects (>40 msec).

### 3.5. Correlations between Activation Latencies and Both Coordination and Functional Walking

There was a good and significant correlation between impaired knee flexion agonist muscle activation latency (LHB) and both FM coordination and the 6MWT functional walking index. For task 1, the correlations were as follows: LHB activation latency and FM: *r* = −  .73 (*P* = .0001); LHB activation latency and 6MWT: *r* = −  .621 (*P* = .0001). For task 2, the correlations were as follows: LHB activation latency and FM: *r* = −  .63 (*P* = .0001); LHB activation latency and 6Min: *r* = −  .56 (*P* = .002).

In contrast, there was poor to no correlation between deactivation latencies (for either agonist or antagonist muscle) and FM coordination and 6 MWT functional walking index. Deactivation correlations ranged from −.03 to −.32 (*P* values ranged .13 to .87).

## 4. Discussion

We found that activation latencies of knee flexors and extensors were abnormally prolonged in subjects with hemiparesis compared with control subjects. These findings in the current study are similar to those reported by others for upper limb muscles [24, 25] and ankle muscles [23]. And the value of activation latencies are similar; for example, our mean activation onset value for normal subjects for knee flexors was 200–239 ± 81 msecs, and Smith et al. [23] reported that mean activation onset for the normal limb ankle muscles was 270 msecs. Others reported significant differences between normal activation latencies and stroke subjects for ankle muscle activations [23]. Our findings extend the literature by describing significant differences between control versus stroke subjects for both knee flexor and knee extensor muscle activation onsets during a ballistic, single joint knee flexion movement.

According to the disablement model [26, 27], the pathway to disability occurs through the sequence of impairment to dysfunction. Others have shown that for stroke, relationships exist between some impairments and some disability model components. The results of the current study extend the literature by identifying relationships among muscle-activation latencies, impairment of joint movement coordination, and walking dysfunction. There was a moderately high and significant correlation between delay in muscle activations and both impairment in joint movement coordination and the functional walking index of 6MWT. These findings suggest that it is critical to address in treatment any delayed muscle activations and deactivations for those stroke patients with chronic gait deficits. 

### 4.1. Muscle Activation Latencies

Abnormally slow muscle activation can present a problem, especially when the safety of the task demands rapid movement and generation of force, as is true for responding to a balance challenge or during the swing phase of gait. Some lower limb movements occur within 250 msecs during walking at normal speeds [28]. The hemiparetic subjects in this study, with swing phase limb flexion impairment, required 723 ± 495 msecs to activate knee flexors during a single joint knee flexion movement in an unweighted condition. Individuals, who could not rapidly flex the limb during the swing phase of gait, had an abnormal and unsafe gait pattern. Buurke et al. [29] found that abnormal muscle activation timings did not recover in stroke survivors tested at 3, 6, 12, and 24 weeks after stroke. Their findings indicated that conventional rehabilitation is not accurately or efficaciously targeting abnormal muscle activation coordination. The persistence of impaired coordination of muscle-activation timing after rehabilitation, provides a potential reason for the dearth of evidence of gait recovery [30] even in the presence of many available methods that are currently used or tested for gait training [30]. Given the relationship of coordinated activation timings to coordinated movements and functional gait, it is critical to develop improved methods to treat muscle activation timing during rehabilitation.

### 4.2. Consistency of Muscle Activation Latencies

The consistency or reliability of knee muscle activation latencies across repetitive movements could have a bearing on whether the knee flexion movement pattern can be used functionally in the swing phase of gait [31]. In the current study, for the control subjects, consistency of activation latencies ranged from 58 to 96 msecs whereas for the hemiparetic subjects, consistency ranged from 94 to 194 msecs. This suggests an inability to normally, reliably control the timing precision of limb flexion. That is, knee joint muscles were not activated consistently, according to intention and expectation. Lack of precision of limb flexion timing, during the swing phase of gait, can result in falls. With poor consistency in muscle activation latency during swing phase [31], a stroke survivor might rely on the more predictable, but less energy-efficient, stifflegged gait compensatory strategy observed after stroke [32, 33].

### 4.3. Close Coupling of Agonist/Antagonist Muscle Activations

In healthy adults, it has been shown that for active knee movement, coactivation occurs for agonist and antagonist muscles [34–37]. The task in the current study involved a rapid movement against no added load and relatively low-friction. For such a movement, the activations of agonist and antagonist muscles are tightly coupled in order to control the speed and quality of the ballistic movement, as borne out in this study for the control subjects. In contrast, the hemiparetic subjects in the current study displayed an abnormally long lag time between knee flexor agonist onset and knee extensor antagonist onset. Decoupled agonist/antagonist pairs at one joint could produce movement that exhibits abnormal variation in velocity during the movement (jerkiness of movement). Uncontrolled speed variations during single joint movement can, in turn, result in impaired coordination of the timing of movements among joints of a single limb or between limbs.

### 4.4. Study Limitations

Stroke results in a broad array of symptoms. This study was focused exclusively on knee joint muscles and the coordination of muscle activations. It was beyond the scope of this study to relate knee joint muscle activation latencies to muscle activation latencies occurring at the hip and the ankle. This will be important work for the future, because the gait pattern demands coordination among muscles at all three lower limb joints. Second, the sample size for the current study did not allow exploration of the difference in muscle activation impairment, according to stroke infarct location or size. Third, stroke survivors younger than 40 were not studied. Finally, it was beyond the scope of this study to correlate the activation latencies of these statically measured latencies with the latencies occurring during the gait pattern. This is important future work.

## 5. Conclusions

Muscle activation and deactivation latencies were significantly longer for hemiparetic subjects versus controls for two knee flexor motor tasks. The delayed activation latencies were significantly correlated with isolated joint coordination movements of the lower limb and with functional walking. Consistency of muscle activation latency was significantly impaired for hemiparetic subjects versus controls. Timing of the coupling of agonist/antagonist muscle activation latencies was impaired in the hemiparetic subjects versus controls. Taken together, these results provide evidence that abnormal muscle timing characteristics after stroke are associated with joint movement coordination impairment and walking disability. Since these impairments and disabilities persist after conventional gait training and rehabilitation, it is critical to develop methods to more accurately target treatment of abnormal muscle activation and deactivation latencies and dyscoordination.

## Figures and Tables

**Table 1:** Subject characteristics.

A. Group	B. Stroke Type		C. Years after Stroke		D. Age Range		E. Gender	
	Ischemic	Hemorrhagic	1-2	>2	40–54	≥55	Male	Female
Stroke	24	3	11	16	7	20	20	7

Controls					2	7	6	3
